# High-throughput sequencing insights into T-cell receptor repertoire diversity in aging

**DOI:** 10.1186/s13073-015-0242-3

**Published:** 2015-11-19

**Authors:** Jörg J. Goronzy, Qian Qi, Richard A. Olshen, Cornelia M. Weyand

**Affiliations:** Division of Immunology and Rheumatology, Department of Medicine, Stanford University School of Medicine, Stanford, CA 94305 USA; Department of Medicine, VAPAHCS, Palo Alto, CA 94306 USA; Department of Biomedical Data Science, Stanford University School of Medicine, Stanford, CA 94305 USA

## Abstract

Decline in T-cell generation leading to T-cell receptor repertoire contraction is a cornerstone of immune system aging, and consequent disorders. High-throughput sequencing enables in-depth immune repertoire characterization, but blood samples are too small to capture its total diversity. New computational models could enable accurate estimation of this diversity.

## The importance of immune repertoire diversity

Among the many biological features of the aging process, the decline in regenerative capacity is perhaps the most obvious [1]. In the immune system, the thymus, the only organ that generates T cells, begins involution with puberty. Under normal physiological conditions, T-cell production after mid-adulthood is minimal. Production of B cells, although active throughout life in the bone marrow, also declines with aging. This lack of regenerative capacity of lymphocytes has been proposed to be a major roadblock to healthy aging, with immune aging contributing to the increased incidence of cancer, and to the increased morbidity and mortality that result from infections, which are due to reduced immune reactivity [2]. In addition, vaccination has only been moderately successful in the elderly. As a consequence of declining regenerative capacity, T- or B-cell repertoires may no longer include sufficient numbers of cells with appropriate receptor specificities to mount a response to infection in this age group [2].

With thymic activity declining, T cells are maintained by homeostatic proliferation of existing T cells, which means that their diversity cannot be increased. T-cell clonal sizes are probably initially similar across all T-cell clones owing to equal initial intrathymic and peripheral clonal expansion, but become more variable with aging, as homeostatic proliferation of naïve T cells is shaped by selective forces [3]. Some T-cell clones might even undergo extinction, thereby leading to contraction of the T-cell repertoire. Loss of clonal specificity is of biological relevance if the repertoire is small, as it impairs the ability of the system to respond to the universe of foreign antigens. In addition to uneven homeostatic proliferation of naïve T cells, the increasing accumulation of memory T cells can have a negative effect on the repertoire of naïve T cells. In chronic or latent infections such as cytomegalovirus, the memory T-cell repertoire specific for this virus is unique in taking up a disproportionate space in some individuals, a process that is coined memory inflation. If these memory cells compete for the same space as naïve T cells, and if the space is not expandable, then memory inflation could have a major negative effect on the ability of the immune system to respond to new challenges.

Studies in mice have supported the notion that T-cell diversity is important. The number of different antigen-specific T-cell receptors (TCRs) that is present in the naïve T-cell repertoire correlated with the magnitude of the ensuing T-cell response [4]. The naïve repertoire also determines the breadth of the memory T-cell repertoire that is important in controlling chronic infection and preventing escape mutants in viral infections. Therefore, immune health is linked tightly to the diversity of TCRs and being able to estimate this diversity might have important clinical applications in assessing immune competence in aging.

## High-throughput TCR sequencing

With the advances in high-throughput sequencing, we now have tools to generate information on TCR repertoire diversity that could be applied to precision medicine to assess the state of the adaptive immune system [5]. Although immunology as a discipline has made major progress over the past decades, diagnostic applications have been limited to measuring inflammatory markers or identifying antibodies. In particular, we lack biomarkers that enable us to quantify immune competence. If available, such quantification tools would be valuable in numerous clinical areas, such as when tailoring immune suppression in transplantation and autoimmune diseases or when vaccinating immunocompromised patients such as the elderly, HIV-infected patients, or patients who have undergone chemotherapy or stem cell transplantation. Considerable efforts have gone into systems immunology approaches to identify markers that predict the quality of a vaccine response and to elucidate relevant pathways in this response. So far, however, approaches to improve vaccine responses in the elderly have been entirely empirical. If low TCR diversity is the cause of a defective immune response, reconstitution therapy approaches will have to be developed or chemotherapeutic agents will have to be selected that are less T-cell-depleting than the approaches and agents that are currently available. Vaccination strategies will have to be modified to recruit low-affinity and cross-reactive T cells. Conversely, determining vaccination-induced increased B- and T-cell clonality will be a valuable marker to assess the nature and quality of a vaccine response [6].

Studies of immune repertoire diversity are complex. Until a few years ago, available techniques could provide only a superficial view of the repertoire, as they only provided information about TCR gene family or were only able to capture the more frequent clones. TCRs are highly polymorphic heterodimers composed of either α and β or γ and δ chains. Diversity is generated through the combination of gene segments and by insertion and deletion of single nucleotides. The potential diversity of αβ TCRs, defined as the number of receptors with different sequences, or as richness, might reach 10^20^. The total number of T cells can reach 10^12^ in humans. Given these enormous numbers, high-throughput sequencing is required to achieve sufficient sequencing depth to estimate clonal abundance. Current studies to estimate TCR repertoire diversity are based on TCR β-chain sequencing, since TCR αβ pairing can be assessed only at the single-cell level.

The first applications of high-throughput sequencing to determine the influence of age on TCR diversity have been published in the last year. Britanova et al*.* described a contraction in TCR diversity associated with aging [7]. As these analyses were done on a sample of unseparated lymphocytes from peripheral blood, the estimates were confounded by the age-associated decrease in frequencies of naïve T cells in blood samples, which emphasizes the importance of studying purified cell subpopulations. We have analyzed fractionated naïve and memory CD4 and CD8 T-cell populations and have found a threefold to fivefold contraction in richness of naïve populations drawn from individuals older than 65 years when compared with those from individuals younger than 35 years [8]. However, our estimates of richness were higher than the previous estimates [7, 9]; whether the observed contraction is of biological relevance is, therefore, unclear. In addition to richness, the extent of clonality in a given repertoire can also be estimated. Clonality is defined as the probability that a sequence is found in two independent replicates. Therefore, this measure can be used to estimate the degree of clonal expansion, which might have been due to uneven homeostatic expansion in the naïve repertoire or to memory inflation in the memory repertoire. In our analysis, clonality of naïve CD8 T cells was far more sensitive to changes in age than was clonality of naïve CD4 T cells [8]. The individuals who participated in our studies were blood bank donors and therefore highly selected for being healthy. A comparison between these individuals with those who are frail or who have undergone chemotherapy would be interesting. A major remaining challenge in these studies is whether we can validate an estimator of richness and whether this estimator is applicable to different age groups or disease states.

## Current challenges

Approximately half of the 10^12^ T cells typically present in individual humans are naïve T cells, and about 2 % of the total number of T cells circulate in the peripheral blood. Studies of T-cell repertoire diversity therefore face two specific challenges: the repertoire in a blood sample needs to be representative of the global repertoire, and the results need to be extrapolated from a small sample of a few million cells to the entire repertoire.

As regards to the first challenge, for naïve T cells under steady-state conditions it seems reasonable to assume that the circulating T-cell repertoire would reflect the entire repertoire. Whether the same is true for memory and effector T cells that undergo kinetic changes, particularly in response to vaccination and infection, is not as clear. Longitudinal studies of these changes can provide information regarding responses to vaccines and on the immune defects that account for decreased responses in the elderly [6].

As regards to the second challenge, exhaustive sequencing of a human peripheral blood sample cannot capture full TCR repertoire diversity [9]. Several approaches have been proposed by which to extrapolate richness from samples analyzed to the full repertoire [10]. Parametric estimators available under the Poisson abundance model are based on the rather speculative assumption of a clonotype frequency distribution that might be incorrect or that might even change with age. Estimates using this approach might severely underestimate richness, in part also because singletons (sequences that are found only once in a sample) are excluded as they could be potential errors in sequencing. Nonparametric estimators do not involve assumptions on the clonal frequency distribution, but they might also be biased by sample size. We used an incidence-based nonparametric estimator by comparing the presence or absence of particular sequences in replicate samples and calculated an estimate of richness in healthy young adults that is at least one order of magnitude higher than previously published numbers obtained using parametric models [8]. Laydon and colleagues proposed, as an alternative approach, to fit candidate models to successively smaller nested subsamples of the actual data and then choose the best performing model to extrapolate the estimate to the real population size [10]. The key assumption in this approach is that there is an asymptote to estimated diversity, and that one can reach this asymptote by studying samples decreasing in size. All of these approaches have their inherent challenges and pitfalls, and the computational estimation of TCR repertoire richness is still a work in progress. By contrast, the approach to estimate clonality is well supported; clonality estimates are reliable and help to identify the defects that compromise vaccine responses in many elderly individuals and the strategies to overcome them.

## Abbreviation

TCR: T-cell receptor
